# A critically ill adolescent with EBV-associated hemophagocytic lymphohistiocytosis-induced hyperinflammatory shock: a case report and literature review

**DOI:** 10.3389/fimmu.2025.1749172

**Published:** 2026-01-12

**Authors:** Yong Chen, Ruodai Zhang, Chenghu Wang, Yi Ren

**Affiliations:** Department of Classical Chinese Medicine, Chongqing Hospital of Traditional Chinese Medicine, Chongqing, China

**Keywords:** adolescent, case report, critical care, Epstein-Barr virus, hemophagocytic lymphohistiocytosis, immunosuppressive therapy, sepsis

## Abstract

Epstein-Barr virus (EBV) infection can trigger life-threatening complications, including hemophagocytic lymphohistiocytosis (HLH) and septic shock. The overlapping clinical manifestations of these conditions pose significant diagnostic and therapeutic challenges. This article reports the case of a previously healthy 19-year-old female who presented with persistent high fever (40.1°C), tachycardia, tachypnea, and hypotension, along with markedly elevated inflammatory markers, meeting the diagnostic criteria for septic shock. Laboratory investigations revealed rapid, significant decreases in white blood cell (WBC) count and platelet count. A positive serum EBV viral capsid antigen (VCA) IgM test led to a suspicion of EBV-associated HLH (EBV-HLH). Prior to a definitive diagnosis of EBV-HLH, the patient was initiated on early and adequate combination therapy with corticosteroids and antivirals. This intervention resulted in rapid clinical and laboratory improvement: body temperature normalized by day 3, and peripheral blood counts gradually returned to normal. The subsequent bone marrow aspiration confirmed the HLH diagnosis, supported by elevated serum ferritin and soluble interleukin-2 receptor (sCD25) levels, fulfilling the diagnostic criteria for HLH. The patient was discharged on a tapering regimen of oral methylprednisolone and achieved complete recovery without relapse during follow-up. This case suggests that rapidly progressive and significant leukopenia and thrombocytopenia are key early indicators for distinguishing EBV-HLH from sepsis alone. Early recognition of EBV-HLH and timely initiation of combined corticosteroid and antiviral therapy can effectively control cytokine storm and improve clinical outcomes in patients with favorable treatment responses, providing valuable clinical insights for managing similar severe cases.

## Introduction

Epstein-Barr virus (EBV), a globally prevalent herpesvirus, exhibits a seroprevalence rate exceeding 90% in adult populations, indicating its extensive infection ubiquity (1). Primary EBV infection in children is often asymptomatic or manifests with non-specific symptoms, whereas in adolescents or adults it typically presents as infectious mononucleosis. Although most EBV infections are self-limiting, they can trigger life-threatening complications in specific individuals. Notably, EBV-associated hemophagocytic lymphohistiocytosis (EBV-HLH) represents a particularly severe and critical condition (2).

EBV-HLH, a hyperinflammatory syndrome, is pathologically characterized by impaired cytotoxic function leading to immune dysregulation and the onset of a cytokine storm (3–5). This process is characterized by excessive activation and proliferation of T lymphocytes and macrophages, accompanied by a massive release of inflammatory factors, ultimately culminating in multiple organ dysfunction syndrome (MODS) (5). Concurrently, the severe immune dysregulation induced by EBV infection predisposes patients to sepsis, which can rapidly progress to septic shock.

The differentiation between hemophagocytic lymphohistiocytosis (HLH) and sepsis is particularly challenging in clinical practice because of their extensive overlap in clinical features, including persistent high fever, organ dysfunction/shock, and a hyperinflammatory state (6). However, their underlying pathologies differ: sepsis primarily stems from an infection-triggered imbalance between anti-inflammatory and pro-inflammatory responses, whereas HLH is characterized by uncontrolled immune activation. Consequently, accurate discrimination between these two entities is crucial for administering targeted therapies, such as immunosuppression.

We report a critical case of an adolescent with EBV infection that progressed to septic shock and fulfilled the diagnostic criteria for HLH. This case clearly demonstrates the complex clinical interplay between these two entities. Successful management was achieved through early recognition of HLH features and the timely initiation of immunosuppressive therapy. By integrating recent advances in the diagnosis and management of EBV-HLH (2, 7) with a deeper mechanistic understanding of the cytokine storm (3, 4), this report aims to provide clinicians with crucial diagnostic insights and therapeutic strategies for managing such critical emergencies.

## Case presentations

Informed consent was obtained from the participants or their legal representatives.

## Case report

We report the case of a previously healthy 19-year-old female who was admitted to the hospital due to a persistent high-grade fever for four days. On admission, clinical evaluation revealed an acutely ill appearance with vital signs indicating hyperthermia (40.1°C), tachycardia (134 bpm), tachypnea (22 breaths/min), and hypotension (87/55 mmHg), consistent with the hemodynamic profile of septic shock. Physical examination showed no hepatosplenomegaly, lymphadenopathy, skin rash, or focal neurological signs. Laboratory tests demonstrated significantly elevated inflammatory markers, including C-reactive protein (CRP), procalcitonin (PCT), and erythrocyte sedimentation rate (ESR), while liver and kidney function, lipid profiles, and electrolyte levels were within normal ranges. Computed tomography from head to pelvis revealed no identifiable infectious focus. Based on these findings, a preliminary diagnosis of septic shock (8) due to suspected community-acquired bacterial infection was established. The patient immediately received aggressive fluid resuscitation to stabilize hemodynamics, empirical antibiotic therapy with cefoperazone-sulbactam, and Xuebijing injection to modulate the excessive inflammatory response.

Notably, repeated blood tests the following day showed a progressive and severe decline in white blood cell and platelet counts. This finding, in conjunction with a positive serological test for Epstein–Barr virus viral capsid antigen (VCA) IgM antibodies, supported the diagnosis of hemophagocytic lymphohistiocytosis (HLH) triggered by primary EBV infection, complicated by septic shock. The treatment strategy was promptly adjusted to include intravenous dexamethasone to control the cytokine storm and ganciclovir as antiviral therapy. To confirm the HLH diagnosis, bone marrow aspiration and biopsy were performed simultaneously, along with tests for serum ferritin, soluble CD25 (sCD25), NK cell activity, and high-throughput microbial gene sequencing.

By the third hospital day, the patient’s body temperature had normalized, and peripheral blood cell counts showed a steady increasing trend. Subsequent diagnostic results fully confirmed the HLH diagnosis (2, 9): bone marrow morphology demonstrated characteristic HLH, serum ferritin and sCD25 levels were significantly elevated, and high-throughput pathogen gene sequencing confirmed the presence of EBV-DNA in the blood. Although NK cell activity remained within the normal range, the overall clinical and laboratory findings satisfied the diagnostic criteria for HLH. With continued glucocorticoid therapy, the patient’s condition improved markedly, and she was discharged. During follow-up, her hematological parameters remained normal, and she received a four-week tapering regimen of oral methylprednisolone. The patient eventually achieved complete recovery and returned to school, with no recurrence observed during follow-up.

**Figure 1:**
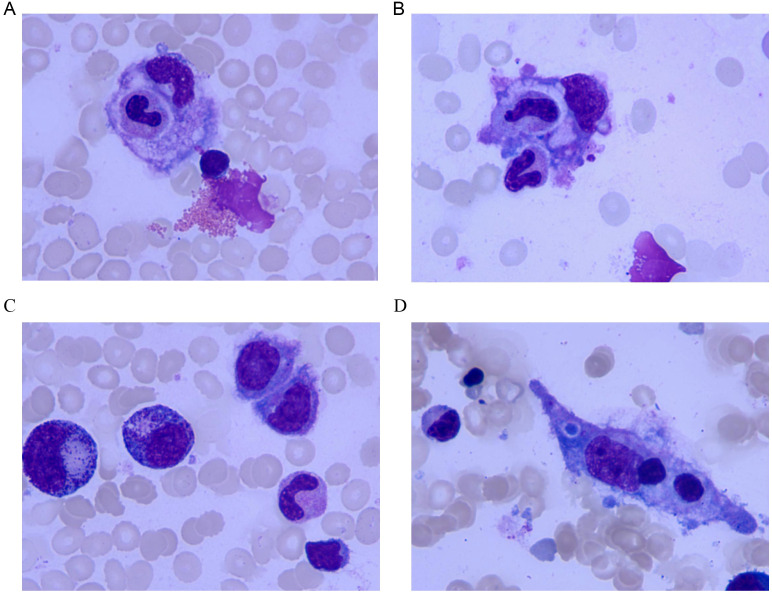
Morphology of hemophagocytic histiocytes in bone marrow aspirate smears (wright-giemsa stain, ×1000). **(A)** Large hemophagocytic histiocyte with light purple cytoplasm containing phagocytosed hematopoietic cellular components, showing the typical morphology of HLH. Numerous mature erythrocytes are present around. **(B)** Multinucleated hemophagocytic histiocyte with multiple irregular dark purple nuclei and light purple cytoplasm containing phagocytic components, reflecting the multinuclear feature of hemophagocytes. **(C)** Hemophagocytic histiocyte on the right (irregular nuclear shape, cytoplasm with phagocytic components), supporting the morphological evidence of HLH. **(D)** Spindle-shaped hemophagocytic histiocyte with light purple cytoplasm phagocytosing multiple dark purple cellular components, demonstrating the morphological diversity of hemophagocytes.

**Figure 2:**
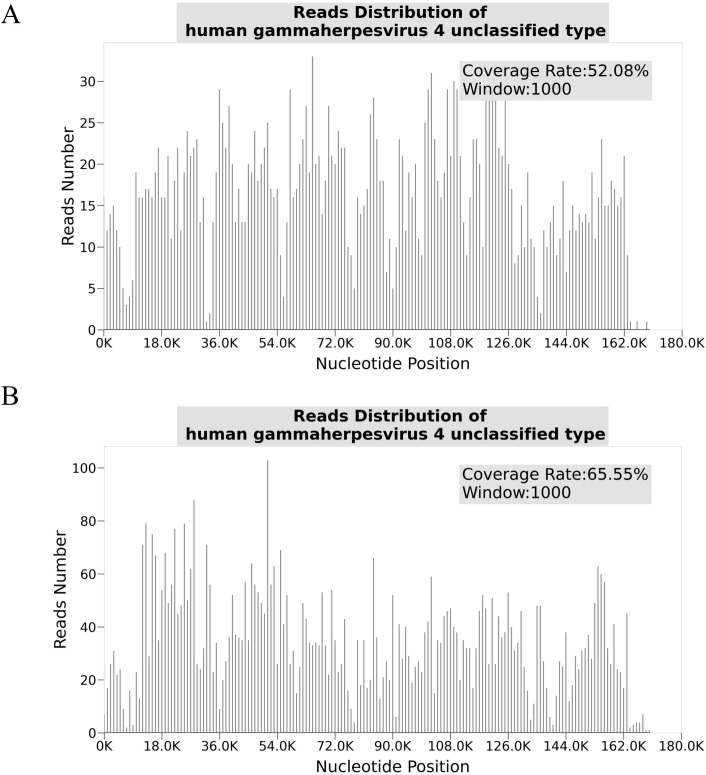
Distribution of high-throughput sequencing reads for human gammaherpesvirus 4 (EBV) in the patient sample. **(A)** Reads the distribution of human gammaherpesvirus four unclassified type with a coverage rate of 52.08% and a window size of 1000. The x-axis represents nucleotide positions (bp), and the y-axis represents the number of reads. **(B)** Reads the distribution of human gammaherpesvirus four unclassified type with a coverage rate of 65.55% and a window size of 1000. Both figures demonstrate EBV genomic coverage and read abundance, confirming its presence in the patient sample.

**Figure 3:**
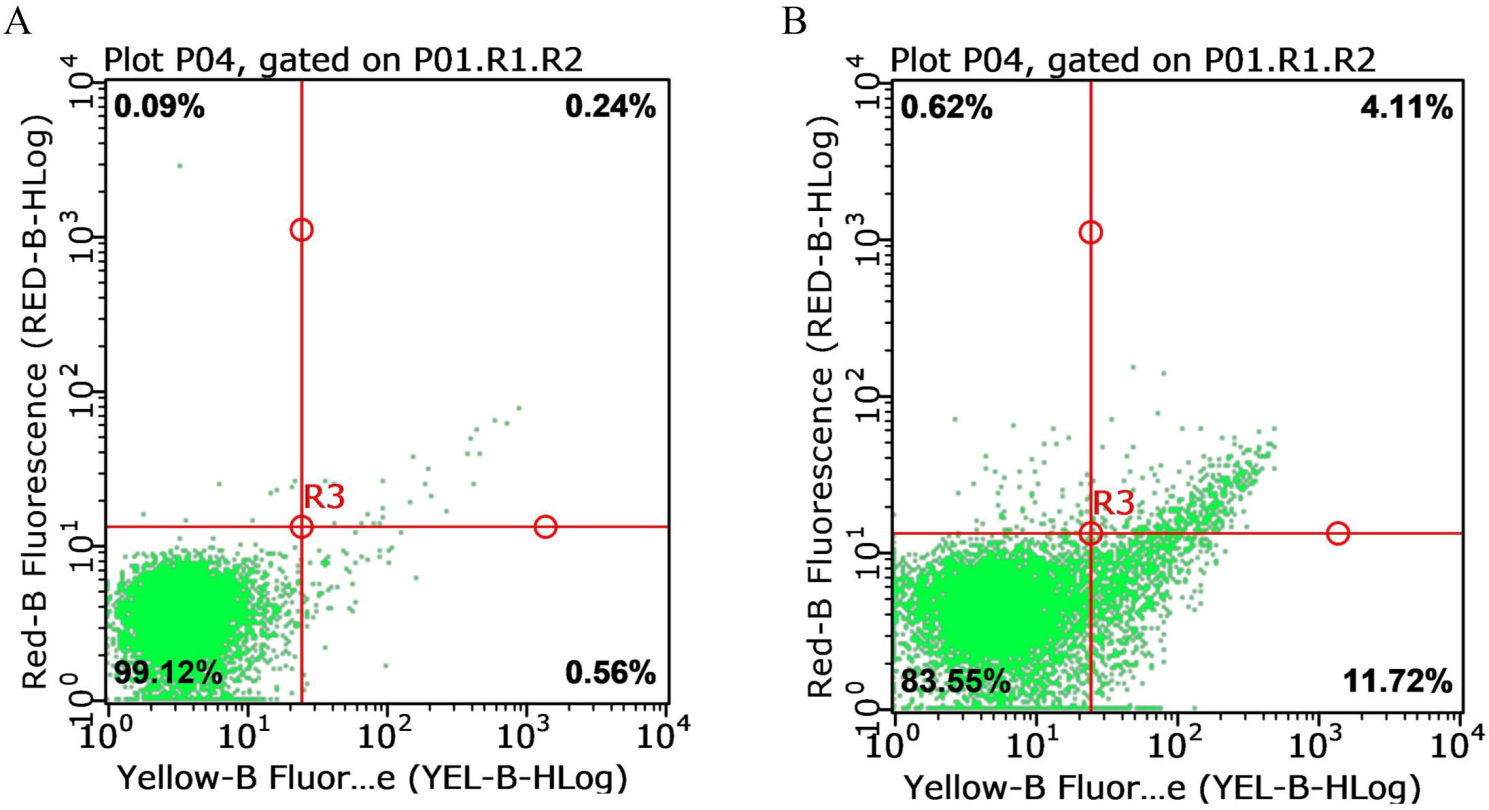
NK cell-mediated cytotoxicity against fluorescent-labeled target cells detected by flow cytometry. **(A)** Spontaneous Apoptosis Background of Target Cells Lower-left quadrant (99.12%): Viable target cells with low yellow and red fluorescence signals, indicating minimal spontaneous apoptosis and a high survival rate of target cells. **(B)** Cytotoxicity of NK Cells Against Target Cells. Lower-left quadrant (83.55%): Viable target cells, showing a significant decrease compared to **(A)** (99.12%), directly demonstrating NK cell-induced target cell death. Total cytotoxicity is estimated as 15.83% (11.72% early apoptosis + 4.11% necrosis/late apoptosis). Note: X-axis (yellow fluorescence) identifies target cells; Y-axis (red fluorescence, e.g., PI) identifies apoptotic/necrotic cells. Analysis was performed using the gating strategy “P01.R1.R2” to ensure the target cell population was analyzed.

**Figure 4:**
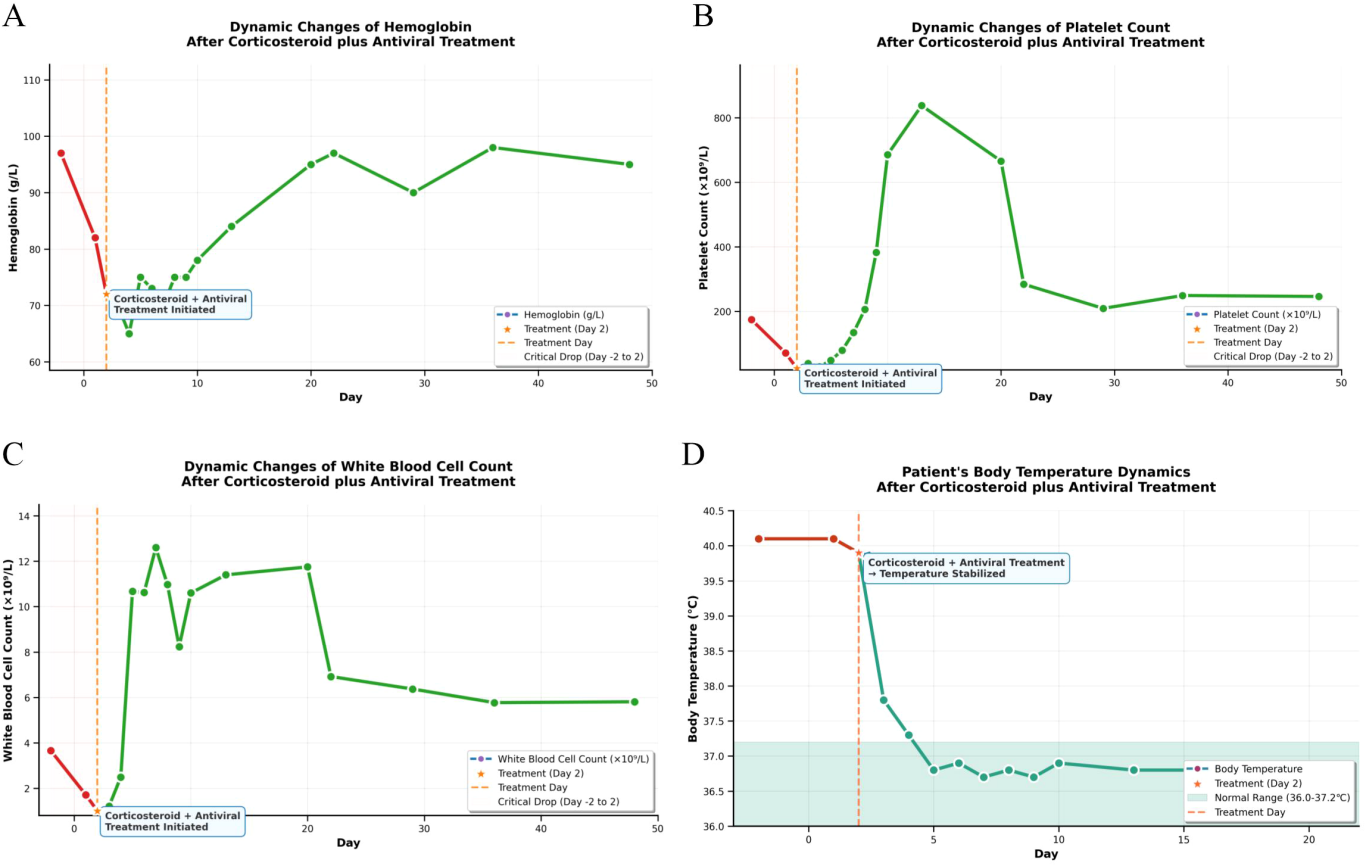
Dynamic changes of key clinical indicators in the patient after corticosteroid plus antiviral treatment​.This figure shows the dynamic trends of the patient’s body temperature and key hematological parameters within 48 days after treatment initiation. **(A)** Hemoglobin level: After admission, the hemoglobin level exhibited a mild downward trend, reached the lowest value on Day 4, then increased steadily, and tended to stabilize after Day 20. **(B)** White Blood Cell (WBC) count: The leukocyte count decreased sharply after admission; following the initiation of corticosteroid plus antiviral treatment on the second day of hospitalization, it gradually increased and remained within the normal reference range from Day 20 onwards. **(C)** Platelet count: The platelet count dropped drastically after admission; after treatment administration on the second day of hospitalization, it gradually recovered, increased rapidly from Day 10, and stabilized within the normal reference range starting from Day 20. **(D)** Body temperature: The patient presented with a persistent high fever (40.1 °C) over the 2 days prior to admission. After treatment initiation on the second day post-admission (marked by an orange star), the body temperature showed a rapid downward trend: it decreased to 37.8 °C on the third day, further dropped to 37.3°C on the fourth day, and stabilized within the normal physiological range (36.0–37.2 °C, indicated by the light green shaded area) from the fifth day onwards.​.

The longitudinal profiles of key laboratory parameters from hospitalization through the post-discharge period (**Table 1**) and the corresponding pharmacological intervention timeline (**Table 2**) are provided in the Supplementary Materials. Critical diagnostic findings demonstrated: bone marrow aspiration confirming hemophagocytic lymphohistiocytosis (HLH) (**Figure 1**), high-throughput sequencing identifying Epstein-Barr virus (EBV) as the etiological agent (**Figure 2**), and preserved natural killer (NK) cell function (**Figure 3**). Upon initiation of corticosteroid and antiviral therapy, the patient rapidly defervesced and subsequently had progressive normalization of peripheral blood counts (**Figure 4**).

**Table 1:** Dynamic changes of laboratory during hospitalization and post-discharg.

Time points	Hematology panel [WBC/Neut#/Eos#/Bas#/Mono#/Lymph#/Hb (g/L)/PLT] (×10^9^/L)	Hepatorenal function [ALT/AST (U/L)/Cr (μmol/L)]	Inflammatory markers [Ferritin/PCT (ng/mL); sCD25 (pg/mL); CRP (mg/L); ESR (mm/h)]	Arterial blood gas [PaO_2_/FiO_2_ Ratio; Lac (mmol/L)]	Lipid metabolism [TG/TC/LDL-C/HDL-C] (mmol/L)	Viral serology [EBV-VCA-IgM] (Result)	Serum electrolytes [K^+^/Na^+^/Cl⁻] (mmol/L)	Coagulation profile [PT (s); INR; TT (s); FIB (g/L); APTT (s)]
Reference Range	4.0–10.0/2.0–7.5/0.02–0.52/0.00–0.10/0.20–1.00/0.80–4.00/120–160/100–300	9–50/15–40/53–106	30–400/<0.05/0-6000/<5/0–15	300–500/0.5–2.2	<1.7/<5.2/<3.4/>1.0	Negative	3.5–5.3/137–147/99–110	11–14/0.8–1.2/12–16/2.0–4.0/25–37
Day -2	3.66/1.99/1.07/0.59/0.00/0.01/97/174	21/26/49	–/0.29/–/–/–	–	–	–	3.67/133/102	–
Day 1	1.71/0.9/0.57/0.23/0.00/0.01/82/71	18/32/43	–/1.4/–/65/21	455/2.8	1.73/2.28/0.35/1.03	+	–	15.7/1.34/17.3/2.8/46.6
Day 2	1/0.43/0.4/0.17/0.00/0.00/72/23	–	581.9/0.93/–/–/–	461/1.2	–	–	–	–
Day 3	1.21/0.39/0.85/0.16/0.00/0.00/70/38	–	–/–/–/58.5/14	541/0.6	–	–	–	15.3/1.31/17.5/2.6/44.8
Day 4	2.49/1.35/0.86/0.28/0.00/0.00/65/28	–	–	–	1.62/–/–/–	–	–	14.8/1.26/17/2.61/34.6
Day 5	10.67/8.35/1.44/0.87/0.00/0.01/75/48	–	–/–/24823/–/–	–	–	–	–	–
Day 6	10.62/8.13/1.74/0.73/0.01/0.01/73/79	–	–	–	–	–	–	–
Day 7	12.6/9.42/2.04/1.02/0.09/0.03/70/134	–	49.9/–/–/8.76/–	–	–	–	3.97/140/105	15.1/1.29/18.3/1.86/36.1
Day 8	10.97/7.82/1.83/1.17/1.14/0.01/75/206	–	–	–	–	–	–	–
Day 9	8.24/5.06/1.75/1.36/0.1/0.02/75/383	–	–	–	–	–	–	–
Day 10	10.6/6.9/2.76/0.97/0.04/0.03/78/686	–	–	–	–	–	–	–
Day 13	11.4/8.16/2.53/0.63/0.03/0.05/84/838	–	–	–	–	–	–	–
Day 20	11.75/7.99/2.77/0.92/0.02/0.05/95/666	–	–	–	–	–	–	–
Day 2 Post-disch.	10.71/6.92/2.45/1.16/0.14/0.05/97/284	–	–	–	–	–	–	–
Day 9 Post-disch.	6.37/3.24/2.45/0.57/0.09/0.02/90/209	–	–	–	–	–	–	–
Day 16 Post-disch.	5.77/3.14/2.04/0.49/0.08/0.02/98/249	–	–	–	–	–	–	–
Day 23 Post-disch.	5.81/3.12/2.06/0.55/0.06/0.02/95/246	–	–	–	–	–	–	–

WBC, White Blood Cell Count; Neut#, Neutrophil Count; Eos#, Eosinophil Count; Bas#, Basophil Count; Mono#, Monocyte Count; Lymph#, Lymphocyte Count; Hb, Hemoglobin; PLT, Platelet; ALT, Alanine Transaminase; AST, Aspartate Transaminase; Cr, Creatinine; PCT, Procalcitonin; sCD25, Soluble Interleukin-2 Receptor; CRP, C-Reactive Protein; ESR, Erythrocyte Sedimentation Rate; PaO_2_/FiO_2_, Arterial Oxygen Partial Pressure/Fraction of Inspired Oxygen; Lac, Lactic Acid; TG, Triglycerides; TC, Total Cholesterol; LDL-C, Low-Density Lipoprotein Cholesterol; HDL-C, High-Density Lipoprotein Cholesterol; EBV-VCA-IgM, Epstein-Barr Virus Viral Capsid Antigen Immunoglobulin M; PT, Prothrombin Time; INR, International Normalized Ratio; TT, Thrombin Time; FIB, Fibrinogen; APTT, Activated Partial Thromboplastin Time; Post-disch., Post-Discharge. "–" indicates the test was not performed or the result was unavailable;"+"indicates the test was positive. Reference ranges are based on the Clinical and Laboratory Standards Institute (CLSI) guidelines for adults.

**Table 2:** Timeline of pharmacological treatment during hospitalization and post-discharge.

Time points	Drug name	Dosage & frequency	Route of administration	Duration
Day 1	Cefoperazone-Sulbactam Inj.	3g,q8h	IV	4days
Day 2	Ganciclovir Inj.	2.5mg,q12h	IV	12days
Day 2	Xuebijing Inj.	100mL,q12h	IV	5days
Day 2	Dex Inj.	10mg,qd	IV	7days
Day 2	rhTPO Inj.	1500IU,qd	SC	5days
Day 2	rhG-CSF Inj.	150ug,qd	SC	3days
Day 2	Esomeprazole EC Caps.	20mg,qd	PO	48days
Day 2	Calcium Carbonate + Vit D3 Tabs.	600mg,bid	PO	48days
Day 2	Leucogen Tabs.	40mg,tid	PO	12days
Day 2	Caffeic Acid Tabs.	0.3g,tid	PO	12days
Day 8	Dex Inj.	5mg,qd	PO	7days
Day 15	MP Tabs.	14mg,qd	PO	7days
Day 2 Post-disch.	MP Tabs.	7mg,qd	PO	7days
Day 9 Post-disch.	MP Tabs.	3.5mg,qd	PO	7days
Day 16 Post-disch.	MP Tabs.	1.75mg,qd	PO	7days
Day 23 Post-disch.	MP Tabs.	0.875mg,qd	PO	7days

IV, Intravenous; PO, Per os (Oral); qd, Quaque die (Once daily); q8h, Quaque 8 hora (Every 8 hours); q12h, Quaque 12 hora (Every 12 hours); Post-disch., Post-discharge; Cefoperazone-Sulbactam Inj., Cefoperazone Sulbactam Injection; Ganciclovir Inj., Ganciclovir Injection; Xuebijing Inj., Xuebijing Injection; Dex Inj., Dexamethasone Injection; rhTPO Inj., Recombinant Human Thrombopoietin Injection; rhG-CSF Inj., Recombinant Human Granulocyte Colony-Stimulating Factor Injection;Esomeprazole EC Caps., Esomeprazole Enteric-Coated Capsules; C alcium Carbonate + Vit D3 Tabs., Calcium Carbonate and Vitamin D3 Tablets; Leucogen Tabs., Leucogen Tablets; Caffeic Acid Tabs., Caffeic Acid Tablets; MP Tabs., Methylprednisolone Tablets.

## Discussion

EBV infection can simultaneously trigger systemic inflammatory responses resembling sepsis and HLH. The significant overlap in their clinical presentations presents a critical diagnostic challenge. In such complex cases, metagenomic next-generation sequencing (mNGS) can rapidly identify EBV, providing crucial clues for early etiological Investigation (10, 11). The emergence of progressively worsening profound leukopenia, particularly in the context of markedly elevated ferritin levels (12, 13), warrants strong suspicion for HLH in the bone marrow over mere inflammatory suppression. In such cases, bone marrow aspiration is diagnostic for confirming HLH. The confluence of these features strongly indicates secondary HLH in the setting of EBV-associated sepsis. A timely and aggressive immunosuppressive regimen aimed at mitigating the cytokine storm is essential and dictates the prognosis following a confirmed diagnosis (9, 14, 15).

Similar to our patient, numerous documented cases of EBV-HLH present with a comparably fulminant and severe disease trajectory. A marked heterogeneity in outcomes is evident across the literature: Yu et al. (16)described a case with refractory disease due to an ITPR3 mutation, necessitating stem cell transplantation; Gu et al. (17) reported a patient with a UNC13D variant and impaired NK-cell function; and Gioia et al. (18) reported a mortality. The successful outcome achieved in our case, therefore, underscores the unique significance of our clinical experience.

The successful management of this case relied on the early and accurate recognition of EBV-associated hemophagocytic lymphohistiocytosis (EBV-HLH) in the clinical setting of septic shock. The therapeutic strategy incorporated fluid resuscitation, broad-spectrum antibiotics, and Xuebijing injection to synergistically modulate the inflammatory response (19–21), supplemented by comprehensive bone marrow function support and meticulous management of complications. The cornerstone of this approach was the early and adequate administration of glucocorticoids to suppress the HLH-associated cytokine storm vigorously.

The therapeutic strategy employed in this case was distinct from the classical HLH-94/2004 protocol or emerging targeted therapies (9, 15). The patient experienced rapid clinical improvement following a regimen centered on high-dose glucocorticoids, combined with antiviral agents and comprehensive supportive care, thereby obviating the need to escalate to agents such as etoposide, cyclosporine A, emapalumab, or ruxolitinib. This approach was guided by two principal considerations: first, glucocorticoids are a cornerstone of HLH induction therapy, capable of potently suppressing the cytokine release storm and lymphocyte hyperactivation; second, growing evidence indicates significant heterogeneity within the HLH disease spectrum. For a subset of patients with non-familial, infection-triggered (e.g., EBV) HLH and without severe underlying immunodeficiency, early and effective control of the hyperinflammatory response with potent anti-inflammatory agents may suffice to avoid the use of cytotoxic drugs or more potent immunosuppressants.

This therapeutic concept is supported by related research. For instance, Qiao et al. (22) successfully managed Severe Fever with Thrombocytopenia Syndrome (SFTS)-associated HLH using methylprednisolone combined with favipiravir, suggesting the potential broad applicability of the “corticosteroid plus antiviral” model for specific virus-induced HLH. Although a study by Zhou et al. (23) confirmed the efficacy of ruxolitinib combined with dexamethasone in newly diagnosed adult HLH patients, it is crucial to note that their cohort consisted solely of individuals requiring systemic therapy, and the regimen underscores the central role of corticosteroids. Collectively, these studies, along with reports from Liu (24), Chandrakasan (25), and Boiten (26), delineate a spectrum of HLH disease: at one end, relatively indolent forms controllable with “corticosteroid plus antiviral” therapy, and at the other, refractory cases necessitating combined targeted agents or monoclonal antibodies. The rapid and favorable response to the “corticosteroid plus antiviral” regimen in our case suggests its position at the more favorable end of this spectrum—notably, this continuum remains an emerging conceptual framework rather than an evidence-based classification system. Furthermore, targeted therapies themselves carry uncertainties, with reports indicating limited efficacy in specific viral contexts, such as measles-associated HLH (27).

Given the substantial overlap in clinical features between HLH-induced shock and septic shock, the diagnosis of this case required a comprehensive evaluation integrating both the Sepsis-3 criteria and the HLH-2004 diagnostic guidelines (2, 8). According to the Sepsis-3 criteria, septic shock is defined by “documented or suspected infection plus a Sequential Organ Failure Assessment (SOFA) score ≥ 2” (8); however, only EBV infection was detected in this case, with no evidence of bacterial or fungal pathogens—representing a critical discrepancy from the core diagnostic requirements of the Sepsis-3 criteria. In contrast, HLH-induced shock is fundamentally an immune dysregulation-driven hyperinflammatory response triggered by an EBV-mediated cytokine storm (3, 4). The patient fulfilled multiple core HLH-2004 diagnostic criteria, including a body temperature of 40.1 °C, neutrophils of 0.43×10^9^/L, platelets of 23×10^9^/L, ferritin of 581.9 ng/mL, soluble CD25 (sCD25) of 24823 pg/mL, and the presence of hemophagocytosis in bone marrow aspirates. Additionally, shock symptoms resolved rapidly following immunosuppressive therapy, further corroborating the diagnosis of HLH-induced shock.

Leukopenia in HLH arises from immune-mediated hematopoietic dysfunction and macrophage-driven hemophagocytosis, with WBC counts typically within the normal range in the early disease phase. In contrast, sepsis-associated leukopenia is primarily attributed to direct suppression of bone marrow hematopoietic stem cells by bacterial toxins, often presenting with elevated WBC counts initially (8, 28). Unlike the rapid, profound cytopenia characteristic of HLH, cytopenia in sepsis progresses gradually over several days to one week, correlating with the duration and severity of infection. In the present case, the white blood cell count declined rapidly from a normal baseline (from 3.66×10^9^/L to 1×10^9^/L within 48 hours), and the patient’s natural killer (NK) cell activity was 15.71% (within the normal reference range). Consistent with the findings of Dou L et al (29), this observation suggests that EBV may induce secondary HLH by impairing NK cell function rather than by reducing NK cell activity. This further confirms that the diagnosis of HLH hinges on a composite assessment of clinical manifestations, laboratory parameters, and therapeutic responses, as a single normal indicator is insufficient to exclude the disease (30). Collectively, these findings underscore inherent uncertainty in the differential diagnosis; nonetheless, we propose a tentative diagnosis of EBV-Associated Hemophagocytic Lymphohistiocytosis-Induced Hyperinflammatory Shock, which aligns with the integrated clinical, laboratory, and therapeutic response data.

The successful reversal of this adolescent patient’s critical condition from EBV-HLH provides a crucial clinical insight. When EBV infection manifests as septic shock accompanied by profound leukopenia, clinicians must transcend the conventional paradigm of “severe infection” and maintain high vigilance for underlying HLH. Profound WBC count and platelets. Serves as the most prevalent and cardinal early features in a subset of HLH cases, necessitating immediate diagnostic evaluation, including ferritin, sCD25 testing, and bone marrow aspiration. Early recognition and decisive initiation of immunosuppressive therapy - rather than merely intensifying anti-infective regimens - represents the critical intervention that can reverse the cytokine storm and be life-saving.

This study has several inherent limitations. First, as a single-case report, its findings do not support the generalizability of the conclusions. The favorable outcome achieved may have been influenced by factors specific to this patient, such as his unique immunological status and the particular virulence of the infecting EBV strain. Second, the diagnosis of HLH was primarily based on clinical criteria and laboratory biomarkers; due to the urgency of the clinical situation, genetic sequencing was not performed to rule out an underlying genetic predisposition definitively. Third, while the combined corticosteroid and antiviral therapeutic regimen used in this case has demonstrated efficacy in EBV-HLH cases with a favorable treatment response, it is not a substitute for the standard treatment protocol centered on corticosteroid-etoposide combination therapy.

In conclusion, a rapid decline in WBC and platelet counts within a short period is an early feature in a subset of EBV-HLH. Early and adequate “corticosteroid plus antiviral” therapy can act as an effective therapeutic test window for patients with this disease, with the core goal of rapidly suppressing its key pathological mechanism—the cytokine storm. If the patient’s body temperature gradually returns to normal during this phase and parameters such as WBC, platelets, serum ferritin, and inflammatory markers show a trend of rapid improvement, escalation to more potent immunosuppressive regimens may be avoided.

## Data Availability

The original contributions presented in the study are included in the article/supplementary material. Further inquiries can be directed to the corresponding authors.
